# Regulation of Matrix Metalloproteinase-2 Secretion from Scleral Fibroblasts and Retinal Pigment Epithelial Cells by miR-29a

**DOI:** 10.1155/2017/2647879

**Published:** 2017-05-29

**Authors:** Yingjie Zhang, Dan-Ning Hu, Yi Zhu, Hao Sun, Ping Gu, Dongqing Zhu, Jibo Zhou

**Affiliations:** ^1^Department of Ophthalmology, Shanghai Ninth People's Hospital, Shanghai Jiaotong University School of Medicine, Shanghai, China; ^2^Departments of Ophthalmology and Pathology, New York Eye and Ear Infirmary of Mount Sinai, New York, NY, USA

## Abstract

**Purpose:**

To identify an effective method to prevent myopia progression by characterizing the regulation of matrix metalloproteinase- (MMP-) 2 expression and its secretion from scleral fibroblasts and retinal pigment epithelium (RPE) cells by miR-29a.

**Methods:**

The effects of miR-29a on the growth of scleral fibroblasts and RPE cells were assessed using the cell counting kit-8. The changes in MMP-2 mRNA levels in scleral fibroblasts and RPE cells after transfection with miR-29a mimics or inhibitor were measured by quantitative PCR. Enzyme-linked immunosorbent assays were used to determine the changes in MMP-2 secretion from scleral fibroblasts and RPE cells after transfection with miR-29a mimics or inhibitor.

**Results:**

The miR-29a mimics or inhibitor did not significantly alter the growth of scleral fibroblasts or RPE cells at 24, 48, or 72 hours after transfection. MMP-2 mRNA levels were significantly decreased in scleral fibroblasts and RPE cells transfected with the miR-29a mimics. The secretion of MMP-2 by scleral fibroblasts and RPE cells was significantly decreased in cells transfected with the miR-29a mimics.

**Conclusions:**

Suppression of scleral fibroblast and RPE cell expression and secretion of MMP-2 by miR-29a can be used as a therapeutic target for the prevention and treatment of myopia.

## 1. Introduction

Myopia is a major public health problem worldwide and is also the leading cause of visual impairment [1]. Determination of the underlying pathogenesis and identification of an effective method to prevent myopia progression are therefore of high priority [2].

The sclera undergoes several changes during the development and progression of myopia, including scleral thinning and weakening [3]. The structural and biomechanical changes in the myopic sclera of human eyes have been well-documented. Besides being thinner than normal, its glycosaminoglycan and collagen contents are reduced and its fibril assembly disorganized, rendering it weaker biomechanically [4–6]. The growth and refractive state of the eye can be manipulated by controlling imposed retinal defocus [7]. The decrease in the retinal pigment epithelial (RPE) cell density was associated with a longer axial length [8]. Therefore, studies of scleral fibroblasts and RPE cells are important for assessing the occurrence and progress of myopia.

Matrix metalloproteinases (MMPs) comprise a family of zinc-dependent endopeptidases that degrade extracellular matrix proteins. More than 20 MMP family members have been identified in humans [9]. Compared with control eyes, MMP-2 is increased in the sclera of myopic eyes induced by form deprivation in chicks [10–12]. Increased scleral MMP-2 expression in form-deprivation myopia has been shown in tree shrews at the protein [13] and mRNA levels [14, 15] and in guinea pigs at the protein level [16]. Modulation of extracellular matrix turnover by altered changes in the RPE secretion of MMPs may play an important role in the normal function and pathology of the retina [17], and because of its location, the RPE likely plays a role in local scleral growth regulation [18].

Micro(mi)RNAs constitute a novel class of short, endogenous noncoding RNAs in animals and plants [19, 20]. miRNAs regulate the translation of specific protein-coding genes by binding to specific regions of the target mRNA, leading to degradation of the mRNA or inhibition of translation [20, 21]. Specifically, the miRNA-29 (miR-29) family consists of an miR-29a/b1 cluster in one chromosome and an miR-29b2/c cluster in a different chromosome. Three online programs, miRanda (http://www.microrna.org/microrna/home.do), TargetScan (http://www.targetscan.org), and TarBase (http://microrna.gr/tarbase), in combination with previous reports were used for predicting miRNAs that might target MMP-2 [22]. miR-29a suppresses the expression and secretion of MMP-2 in various cancers. 3′-UTR luciferase reporter assay data imply that miR-29a attenuates the expression of MMP-2 by targeting the MMP-2 3′-UTR directly [22]. However, little is known regarding the effects of miR-29a on the expression and secretion of MMP-2 by human scleral fibroblasts and RPE cells.

Therefore, this study characterized the regulation of MMP-2 expression in, and secretion from, scleral fibroblasts and RPE cells by miR-29a.

## 2. Materials and Methods

### 2.1. Ethics

Informed consent was provided in accordance with the Declaration of Helsinki. The study was approved by the Shanghai Jiao Tong University School of Medicine Ethics Review Board and the Ethics Committee of Shanghai Ninth People's Hospital.

### 2.2. Cell Culture

Scleral fibroblasts (donated by Wenzhou Medical University) and ARPE-19 cells were both cultured in complete medium containing 10% fetal bovine serum (Invitrogen, Carlsbad, CA, USA) and Dulbecco's Modified Essential Medium at 37°C with 5% CO_2_. The medium was changed every 2 days. The cells were passaged using 0.05% trypsin and 0.02% EDTA (Life Technologies, Gaithersburg, MD, USA).

### 2.3. MiRNA Transfection

MiR-29a oligonucleotides included miR-29a mimics (miR-29a precursor molecules that possess structures similar to that of the intracellular miR-29a precursor but have been chemically modified and optimized to enable processing into mature miRNAs by mimicking the miR-29a natural shearing process) and an miR-29a inhibitor, all synthesized by Biomics Biotech (Nantong, China). The sequences of the miR-29a mimics were 5′-UAGCACCAUCUGAAAUCGGUUA-3′ and 5′-UAACCGAUUUCAGAUGGUGCUA-3′. Transfection was performed using Lipofectamine® 2000 (Invitrogen), in accordance with the manufacturer's instructions.

### 2.4. Detection of miR-29a Expression

Scleral fibroblasts and RPE cells were grown in six-well plates to 60–70% confluence before transfection. The cells in each well of a six-well plate were transfected with 50 nM mimics or 50 nM inhibitor. Total RNA was extracted from scleral fibroblasts and RPE cells after 24 hours transfection with the miR-29a mimics or inhibitor and used for quantitative PCR (qPCR). The PCR primers for miR-29a were 5′-TAGCACCATCTGAAATCG-3′ (forward) and 5′-CACACCAGCACTGACTA-3′ (reverse). Untransfected cells treated with Lipofectamine 2000 were used as negative controls (NCs).

### 2.5. Cell Growth Assays

The effects of miR-29a on scleral fibroblast and RPE cell growth were assessed using a cell counting kit (CCK8; Dojindo, Kumamoto, Japan). Scleral fibroblasts and RPE cells were seeded at a final density of 3 × 10^3^/well and cultured in 96-well plates. After treatment with the miR-29a mimics or inhibitor, the CCK8 solution was added to each well on days 0, 1, 2, and 3 of culturing. After the cells were incubated for another 4 hours at 37°C according to the manufacturer's instructions, the absorbance at 450 nm was measured using a microplate reader (ELx800; BioTek Instruments, Winooski, VT, USA). The viable cell number was directly proportional to the absorbance at 450 nm, and thus viability was expressed as the absorbance at this wavelength.

### 2.6. Total RNA Isolation, Reverse Transcription, and Quantitative Polymerase Chain Reaction (qPCR)

Total RNA was extracted from scleral fibroblasts and RPE cells using TRIzol® reagent (Invitrogen) at 24 and 48 hours after transfection with miR-29a mimics or inhibitor and then analyzed by qPCR. The untransfected cells were used as controls. The extracted total RNA concentration was measured using a spectrophotometer and NanoDrop™ 2000 software (Thermo Scientific, Waltham, MA, USA), and the purity was assessed by OD_260/280 nm_ ratios between 1.9 and 2.1. Then, 1,000 ng quantitated RNA was reverse-transcribed using the PrimeScript™ RT reagent kit (Perfect Real-Time; Takara, Dalian, China). qPCR was performed in a 10 *μ*L total volume containing 5 *μ*L 2x Power SYBR Green PCR Master Mix (Applied Biosystems, Foster City, CA, USA), 1 *μ*L diluted cDNA, and 150 nM gene-specific primers, supplemented with nuclease-free water (Invitrogen). The PCR primers used to amplify glyceraldehyde-3-phosphate dehydrogenase (GAPDH) were 5′-TGAACTGAAAGCTCTCCACC-3′ (forward) and 5′-CTGATGTACCAGTTGGGGAA-3′ (reverse), and those used to amplify MMP-2 were 5′-GCCAAGTGGTCCGTGTGAAGTA-3′ (forward) and 5′-GCCGTACTTGCCATCCTTCTCA-3′ (reverse). The amplification efficiency of the primers was measured using serial dilutions of the cDNA (1 : 1, 1 : 5, 1 : 25, 1 : 125, 1 : 625, and 1 : 3,125). The samples were then amplified using the 7500 Real-Time PCR Detection System (Applied Biosystems). After 40 cycles of amplification, relative mRNA levels were analyzed using the Pfaffl method [23]. The relative mRNA level was expressed as the fold change relative to the negative controls after normalization to GAPDH expression.

### 2.7. Western Blot Analysis

After RPE cells were incubated with either the miR-29a mimics or inhibitor for 12 hours, total cellular proteins were harvested for western blot analyses. A BCA kit (Pierce, Rockford, IL, USA) was used to determine the protein concentrations. A 40 *μ*g protein sample was then subjected to sodium dodecyl sulfate-polyacrylamide gel electrophoresis to separate the proteins. Subsequently, the proteins were transferred to a 0.22 mm polyvinylidene fluoride membrane (Millipore, Billerica, MA, USA). The membranes were then blocked with 5% bovine serum albumin and incubated with rabbit monoclonal anti-MMP-2 at a dilution of 1 : 300 (SAB, MD, USA), followed by mouse anti-*β*-actin at a dilution of 1 : 5,000 (Sigma-Aldrich, St. Louis, MO, USA) overnight at 4°C. After incubation with DyLight™ 680-conjugated secondary antibodies at a dilution of 1 : 5,000 (Sigma-Aldrich), the protein expression levels were determined using the Odyssey V 3.0 image scanner (LI-COR, Lincoln, NE, USA). The intensities of the protein bands were quantified densitometrically using BandScan software, ver. 5.0, and the values for each sample were normalized against *β*-actin.

### 2.8. Enzyme-Linked Immunosorbent Assay (ELISA)

The ELISA kit for MMP-2 detection was purchased from RayBiotech (Atlanta, GA, USA). Scleral fibroblasts and RPE cells were seeded into six-well plates at a final density of 1 × 10^5^/well. The cells were separately transfected with 50 nM miR-29a mimics or 50 nM miR-29a inhibitor. Conditioned culture medium was collected at 48 or 72 hours after transfection and stored at −80°C until analysis. ELISA was performed according to the manufacturer's instructions.

### 2.9. Statistical Analyses

The experimental results were expressed as means ± standard derivation. All experiments were performed in triplicate unless specified otherwise. The data from the experimental groups were compared with those from the controls. Statistical analyses were performed using the one-way analysis of variance (ANOVA) or paired *t*-tests using GraphPad Prism ver. 6.01. A value of *P* < 0.05 was considered to indicate a significant difference.

## 3. Results

### 3.1. Expression of miR-29a in Scleral Fibroblasts and Retinal Pigment Epithelial (RPE) Cells after Transfection

**Figure 1:**
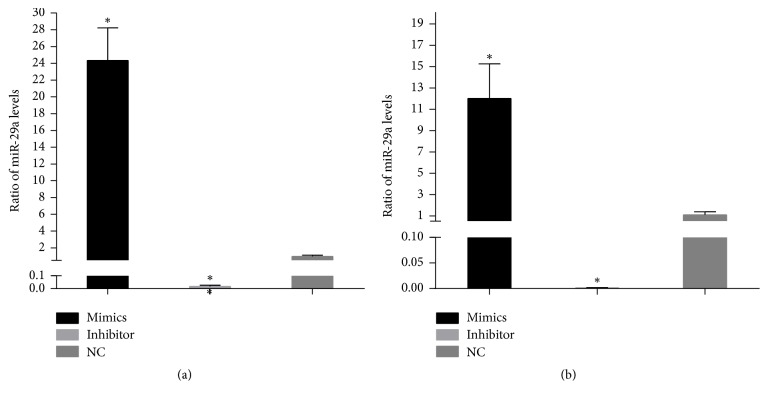
The efficiency of miR-29a transfection of scleral fibroblasts and retinal pigment epithelial (RPE) cells. Scleral fibroblasts and RPE cells were grown in six-well plates to 60–70% confluency before transfection. The cells in each well were transfected with 50 nM miR-29a mimics or 50 nM miR-29a inhibitor. The quantitative PCR results showed that, in scleral fibroblasts (a) and RPE cells (b), miR-29a was significantly upregulated by transfection of the miR-29a mimics and downregulated by transfection of the miR-29a inhibitor compared with negative controls (*P* < 0.05). Note: the error bars show the standard deviations (*n* = 3). The statistical analyses were performed using one-way ANOVA, ^*∗*^*P* < 0.05. NC, negative controls.

The cells were collected 24 hours after transfection of scleral fibroblasts or RPE cells with miR-29a mimics or inhibitor. The qPCR analyses showed that the miR-29a levels were significantly upregulated in scleral fibroblasts and RPE cells after transfection with the miR-29a mimics (*P* < 0.05; Figure 1). In contrast, the levels of miR-29a were significantly downregulated by transfection with the miR-29a inhibitor (*P* < 0.05; Figure 1).

### 3.2. The Effects of miR-29a on the Growth of Scleral Fibroblasts and RPE Cells

**Figure 2:**
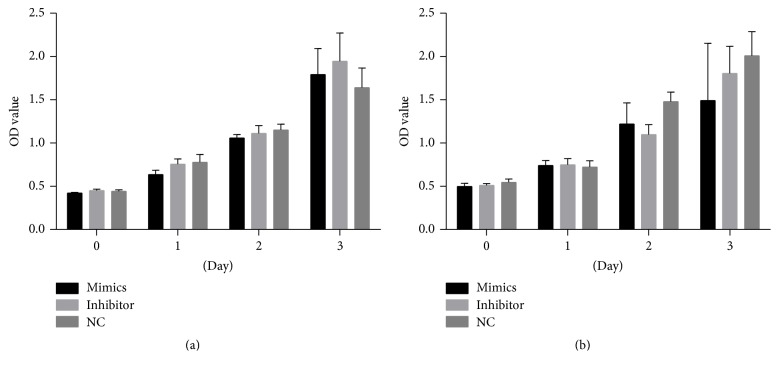
The effects of miR-29a on the viability and growth of scleral fibroblasts and retinal pigment epithelial (RPE) cells. Scleral fibroblasts and RPE cells were seeded at a final density of 3 × 10^3^/well and cultured in 96-well plates. The cells in each well were transfected with 50 nM miR-29a mimics or 50 nM inhibitor. The miR-29a mimics or inhibitor had no significant effect on the growth of scleral fibroblasts (a) or RPE cells (b) at 24, 48, or 72 hours after transfection compared with the negative controls (*P* > 0.05). Note: the error bars show the standard deviations (*n* = 3). The statistical analyses were performed using one-way ANOVA. NC, negative control.

To assess the effects of miR-29a on the cell growth of scleral fibroblasts and RPE cells, the miR-29a mimics or inhibitors were transfected into scleral fibroblasts and RPE cells, and growth was assessed relative to that of the negative control (NC) using the CCK8 kit. miR-29a mimics or inhibitor did not significantly affect the growth of scleral fibroblasts or RPE cells at 24, 48, or 72 hours after transfection compared with the NC (*P* > 0.05; Figure 2).

### 3.3. Changes in MMP-2 mRNA Levels in Scleral Fibroblasts and RPE Cells after Transfection with miR-29a Mimics or Inhibitor

**Figure 3:**
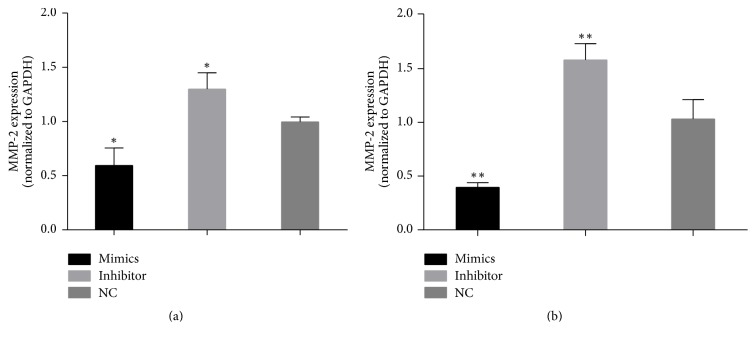
Changes in matrix metalloproteinase- (MMP-) 2 mRNA levels in scleral fibroblasts and retinal pigment epithelial (RPE) cells after transfection with miR-29a mimics or inhibitor. Scleral fibroblasts and RPE cells were grown in six-well plates to 60–70% confluency before transfection. The cells in each well of a six-well plate were transfected with 50 nM mimics or 50 nM inhibitor. Quantitative PCR analyses showed that transfection of the miR-29a mimics decreased MMP2 expression in scleral fibroblasts (a) (*P* < 0.05) and RPE cells (b) (*P* < 0.01), while the inhibitor increased MMP-2 expression in scleral fibroblasts (a) (*P* < 0.05) and RPE cells (b) (*P* < 0.01) 24 hours after transfection compared with negative controls. Note: the error bars show the standard deviations (*n* = 3). The statistical analyses were performed using one-way ANOVA. ^*∗*^*P* < 0.05, ^*∗∗*^*P* < 0.01. NC, negative control.

To characterize the possible relationships between the expression of MMP-2 and miR-29a, miR-29a mimics or inhibitors were transfected into scleral fibroblasts and RPE cells. qPCR analyses showed that MMP-2 mRNA levels were significantly decreased in scleral fibroblasts (*P* < 0.05) and RPE cells (*P* < 0.01) transfected with the miR-29a mimics, and MMP-2 mRNA levels were significantly increased in scleral fibroblasts (*P* < 0.05) and RPE cells (*P* < 0.01) transfected with the miR-29a inhibitor (Figure 3).

### 3.4. Changes in MMP-2 Protein Levels in Scleral Fibroblasts and RPE Cells after Transfection with miR-29a Mimics or Inhibitor

**Figure 4:**
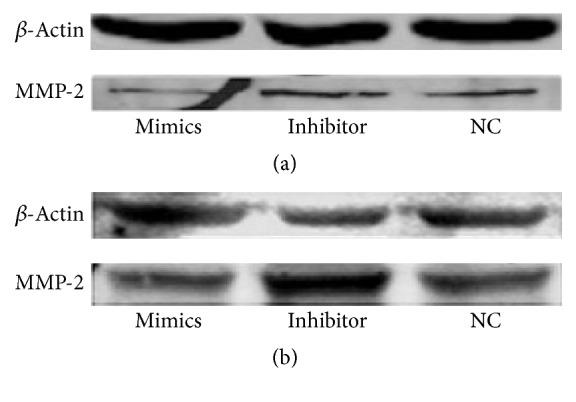
Changes in matrix metalloproteinase- (MMP-) 2 protein levels in scleral fibroblasts (a) and retinal pigment epithelial (RPE) cells (b) after transfection with miR-29a mimics or inhibitor. The scleral fibroblasts and RPE cells were grown in six-well plates to 60–70% confluence before transfection. The cells in each well were transfected with 50 nM mimics or 50 nM inhibitor. Western blots showed that the miR-29a mimics decreased MMP2 expression, and the inhibitor increased MMP-2 expression in both scleral fibroblasts and RPE cells 24 hours after transfection. NC, negative control.

Western blotting was performed to measure the possible changes in intracellular MMP-2 protein expression. The results showed that the miR-29a mimics decreased MMP-2 expression, while the miR-29a inhibitor increased MMP-2 protein expression in both scleral fibroblasts and RPE cells (Figure 4).

### 3.5. Detection of MMP-2 Secretion by Scleral Fibroblasts and RPE Cells after Transfection with the miR-29a Mimics or Inhibitor Using ELISA

**Figure 5:**
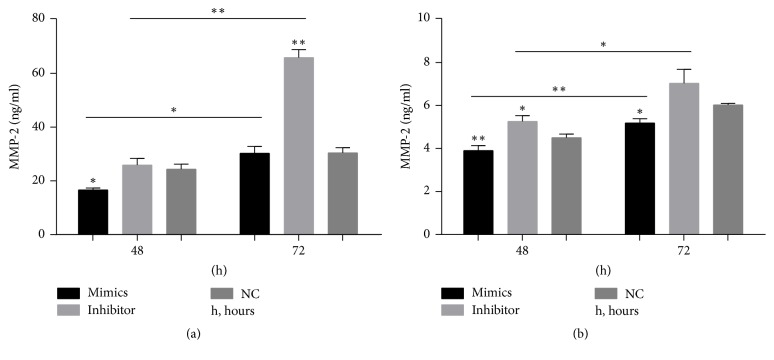
Changes in matrix metalloproteinase- (MMP-) 2 secretion from scleral fibroblasts and retinal pigment epithelial (RPE) cells after transfection with miR-29a mimics or inhibitor. The protein secreted from scleral fibroblasts and RPE cells was analyzed using enzyme-linked immunosorbent assays (ELISAs). The secretion of MMP-2 by scleral fibroblasts after 48 hours was decreased in cells transfected with the miR-29a mimics compared with negative controls (*P* < 0.05). The secretion of MMP-2 by scleral fibroblasts after 72 hours was significantly increased in cells transfected with the miR-29a inhibitor compared with negative controls (*P* < 0.01). The secretion of MMP-2 was higher 72 hours after transfection than 48 hours after transfection in both the miR-29a mimics group (*P* < 0.05) and miR-29a inhibitor group (*P* < 0.01). (a) The secretion of MMP-2 by RPE cells was significantly decreased after 48 (*P* < 0.01) and 72 (*P* < 0.05) hours in cells transfected with the miR-29a mimics compared with negative controls. The secretion of MMP-2 by RPE cells after 48 hours was significantly increased in cells transfected with the miR-29a inhibitor compared with negative controls (*P* < 0.05). The secretion of MMP-2 was higher 72 hours after transfection than 48 hours after transfection in both the miR-29a mimics group (*P* < 0.01) and miR-29a inhibitor group (*P* < 0.05) (b). Note: the error bars show the standard deviations (*n* = 3). The statistical analyses were performed using one-way ANOVA for groups at same time point and paired* t*-tests for between-group analyses at different time points. ^*∗*^*P* < 0.05, ^*∗∗*^*P* < 0.01. NC, negative control; h, hour.

ELISA showed that the secretion of MMP-2 by scleral fibroblasts 48 hours after transfection with the miR-29a mimics was decreased significantly (*P* < 0.05). The secretion of MMP-2 by scleral fibroblasts 72 hours after transfection with the miR-29a inhibitor was increased significantly (*P* < 0.01). The secretion of MMP-2 was higher 72 hours after transfection than 48 hours after transfection in both the miR-29a mimics group (*P* < 0.05) and miR-29a inhibitor group (*P* < 0.01) (Figure 5(a)). The secretion of MMP-2 by RPE cells with miR-29a mimics was decreased 48 (*P* < 0.01) and 72 (*P* < 0.05) hours after transfection. The secretion of MMP-2 by RPE cells 48 hours after transfection with the miR-29a inhibitor was increased significantly (*P* < 0.05). The secretion of MMP-2 was higher 72 hours after transfection than 48 hours after transfection in both the miR-29a mimics group (*P* < 0.01) and miR-29a inhibitor group (*P* < 0.05) (Figure 5(b)).

## 4. Discussion

Previous studies have shown that expression of miR-29 family members is downregulated in a wide range of cancers and that their upregulation suppresses tumor metastasis [24]. Numerous studies have also shown that miR-29s suppresses the expression and secretion of MMP-2 [22, 25–31]. Lu et al. showed that miR-29a mimics decreased MMP-2 expression in human oral squamous cell carcinoma cell line SCC-25 cells, whereas miR-29a inhibitors increased MMP-2 expression in SCC-9 [22]. Targeting MMP-2 by miR-29b is a mechanism whereby American ginseng hexane extract suppresses the migration of colon cancer cells [31]. Moreover, Wang et al. showed that miR-29c inhibited expression of a luciferase gene construct containing the 3′-UTR of MMP-2 mRNA and miR-29c downregulated the expression of MMP-2 at the protein level in lung cancer cell line 95D [29].

In studies of ocular diseases, Cai et al. reported that, during choroidal neovascularization, activation of NF*κ*B inhibits the expression of miR-29 family members. This process might have contributed to angiogenesis by upregulating the MMP-2 protein levels in RPE cells [32]. Moreover, Xie et al. reported that the* miR-29a rs157907*A/G polymorphism was associated with a decreased risk of high myopia in the Chinese population [33].

The results of the present study showed that miR-29a downregulated MMP-2 expression in scleral fibroblasts and RPE cells. These results were consistent with previous reports [22, 26, 32] on the effects of miR-29s on the expression of MMPs. miR-29a had little effect on the growth of scleral fibroblasts and RPE cells. Numerous studies on myopia have emphasized changes in MMP-2 expression in the sclera [10–16, 34]. Jones et al. reported that eye growth induced by retinal-image degradation involved increases in the activities of multiple scleral proteinases that could modify the biomechanical properties of scleral structural components and contribute to tissue remodeling and growth [10]. Jones et al. reported that MMP-2 played important roles in remodeling the extracellular matrix of the sclera and in the development and progression of myopia [10, 13]. Using immunohistochemical analysis, Yang et al. demonstrated that the expression of collagen I was significantly lower and the expression of MMP-2 was significantly higher in the posterior sclera in the defocused eyes compared with the contralateral eyes [16]. Rada et al. reported that TIMP-2 expression was significantly decreased in the posterior sclera of form-deprived eyes [12].

In this study, we examined only the regulation of MMP-2 expression in and secretion from scleral fibroblasts and RPE cells by miR-29a in vitro and did not use animal models to investigate the function of miR-29a in vivo. It is necessary to observe the interference effect of miR-29a on eyeball development and myopia in animal models. The expression of TIMP-2 in and its secretion from scleral fibroblasts and RPE cells were as important as MMP-2 and will be investigated in a future study. Collagen I and IV on the sclera was related to myopia [15, 16]. In a future study, we will determine the expression of collagen I and IV in form-deprived eye sclera and detect the changes in collagen I and IV with miR-29a in vivo.

In conclusion, the suppression of MMP-2 expression and its secretion in scleral fibroblasts and RPE cells stimulated by miR-29a may provide a molecular basis for understanding the mechanisms underlying myopia progression.

## References

[1] Pan C.-W., Ramamurthy D., Saw S.-M. (2012). Worldwide prevalence and risk factors for myopia. *Ophthalmic and Physiological Optics*.

[2] Koffler B. H., Sears J. J. (2013). Myopia control in children through refractive therapy gas permeable contact lenses: Is it for real?. *American Journal of Ophthalmology*.

[3] Jonas J. B., Jonas S. B., Jonas R. A., Holbach L., Panda-Jonas S. (2011). Histology of the parapapillary region in high myopia. *American Journal of Ophthalmology*.

[4] Curtin B. J. (1969). Physiopathologic aspects of scleral stress-strain. *Transactions of the American Ophthalmological Society*.

[5] Avetisov E. S., Savitskaya N. F., Vinetskaya M. I., Iomdina E. N. (1983). A study of biochemical and biomechanical qualities of normal and myopic eye sclera in humans of different age groups. *Metabolic, Pediatric and Systemic Ophthalmology*.

[6] Curtin B. J., Iwamoto T., Renaldo D. P. (1979). Normal and staphylomatous sclera of high myopia. An electron microscopic study. *Archives of Ophthalmology*.

[7] Benavente-Pérez A., Nour A., Troilo D. (2014). Axial eye growth and refractive error development can be modified by exposing the peripheral retina to relative myopic or hyperopic defocus. *Investigative ophthalmology & visual science*.

[8] Jonas J. B., Ohno-Matsui K., Holbach L., Panda-Jonas S. (2017). Retinal pigment epithelium cell density in relationship to axial length in human eyes. *Acta Ophthalmologica*.

[9] Murphy G., Nagase H. (2009). Progress in matrix metalloproteinase research. *Molecular Aspects of Medicine*.

[10] Jones B. E., Thompson E. W., Hodos W., Waldbillig R. J., Chader G. J. (1996). Scleral matrix metalloproteinases, serine proteinase activity and hydrational capacity are increased in myopia induced by retinal image degradation. *Experimental Eye Research*.

[11] Rada J. A., Brenza H. L. (1995). Increased latent gelatinase activity in the sclera of visually deprived chicks. *Investigative Ophthalmology and Visual Science*.

[12] Rada J. A., Perry C. A., Slover M. L., Achen V. R. (1999). Gelatinase A and TIMP-2 expression in the fibrous sclera of myopic and recovering chick eyes. *Investigative Ophthalmology and Visual Science*.

[13] Guggenheim J. A., McBrien N. A. (1996). Form-deprivation myopia induces activation of scleral matrix metalloproteinase-2 in tree shrew. *Investigative Ophthalmology and Visual Science*.

[14] Siegwart J. T., Norton T. T. (2002). The time course of changes in mRNA levels in tree shrew sclera during induced myopia and recovery. *Investigative Ophthalmology and Visual Science*.

[15] Siegwart J. T., Norton T. T. (2001). Steady state mRNA levels in tree shrew sclera with form-deprivation myopia and during recovery. *Investigative Ophthalmology and Visual Science*.

[16] Yang S. R., Ye J. J., Long Q. (2010). Expressions of collagen , matrix metalloproteases-2, and tissue inhibitor of matrix metalloproteinase-2 in the posterior sclera of newborn guinea pigs with negative lens-defocused myopia. *Acta Academiae Medicinae Sinicae*.

[17] Alexander J. P., Bradley J. M., Gabourel J. D., Acott T. S. (1990). Expression of matrix metalloproteinases and inhibitor by human retinal pigment epithelium. *Investigative Ophthalmology and Visual Science*.

[18] Rymer J., Wildsoet C. F. (2005). The role of the retinal pigment epithelium in eye growth regulation and myopia: a review. *Visual Neuroscience*.

[19] Lagos-Quintana M., Rauhut R., Lendeckel W., Tuschl T. (2001). Identification of novel genes coding for small expressed RNAs. *Science*.

[20] Bartel D. P. (2004). MicroRNAs: genomics, biogenesis, mechanism, and function. *Cell*.

[21] Cannell I. G., Kong Y. W., Bushell M. (2008). How do microRNAs regulate gene expression?. *Biochemical Society Transactions*.

[22] Lu L., Xue X., Lan J. (2014). MicroRNA-29a upregulates MMP2 in oral squamous cell carcinoma to promote cancer invasion and anti-apoptosis. *Biomedicine and Pharmacotherapy*.

[23] Pfaffl M. W. (2001). A new mathematical model for relative quantification in real-time RT-PCR. *Nucleic Acids Research*.

[24] Wang H., Garzon R., Sun H. (2008). NF-*κ*B-YY1-miR-29 regulatory circuitry in skeletal myogenesis and rhabdomyosarcoma. *Cancer Cell*.

[25] Fang J.-H., Zhou H.-C., Zeng C. (2011). MicroRNA-29b suppresses tumor angiogenesis, invasion, and metastasis by regulating matrix metalloproteinase 2 expression. *Hepatology*.

[26] Jones J. A., Stroud R. E., O'Quinn E. C. (2011). Selective microRNA suppression in human thoracic aneurysms: relationship of miR-29a to aortic size and proteolytic induction. *Circulation: Cardiovascular Genetics*.

[27] Presneau N., Eskandarpour M., Shemais T. (2013). MicroRNA profiling of peripheral nerve sheath tumours identifies miR-29c as a tumour suppressor gene involved in tumour progression. *British Journal of Cancer*.

[28] Rossi M., Pitari M. R., Amodio N. (2013). miR-29b negatively regulates human osteoclastic cell differentiation and function: implications for the treatment of multiple myeloma-related bone disease. *Journal of Cellular Physiology*.

[29] Wang H., Zhu Y., Zhao M. (2013). miRNA-29c suppresses lung cancer cell adhesion to extracellular matrix and metastasis by targeting integrin *β*1 and matrix metalloproteinase2 (MMP2). *PLoS ONE*.

[30] Fan Y.-C., Mei P.-J., Chen C., Miao F.-A., Zhang H., Li Z.-L. (2013). MiR-29c inhibits glioma cell proliferation, migration, invasion and angiogenesis. *Journal of Neuro-Oncology*.

[31] Poudyal D., Cui X., Le P. M. (2013). A key role of microRNA-29b for the suppression of colon cancer cell migration by american ginseng. *PLoS ONE*.

[32] Cai J., Yin G., Lin B. (2014). Roles of NF*κ*B-miR-29s-MMP-2 circuitry in experimental choroidal neovascularization. *Journal of Neuroinflammation*.

[33] Xie M., Li Y., Wu J., Wu J. (2016). Genetic variants in MiR-29a associated with high myopia. *Ophthalmic Genetics*.

[34] Li X. J., Yang X. P., Wan G. M., Wang Y. Y., Zhang J. S. (2014). Effects of hepatocyte growth factor on MMP-2 expression in scleral fibroblasts from a guinea pig myopia model. *International Journal of Ophthalmology*.

